# Mechanisms of TNF-independent RIPK3-mediated cell death

**DOI:** 10.1042/BCJ20210724

**Published:** 2022-10-14

**Authors:** Bart Tummers, Douglas R. Green

**Affiliations:** 1 Centre for Inflammation Biology and Cancer Immunology, Department of Inflammation Biology, School of Immunology and Microbial Sciences, King's College London, London SE1 1UL, U.K.; 2 Department of Immunology, St. Jude Children's Research Hospital, Memphis, TN 38105, U.S.A

**Keywords:** apoptosis, cell death, immunology, inflammation, necroptosis, RIPK3

## Abstract

Apoptosis and necroptosis regulate many aspects of organismal biology and are involved in various human diseases. TNF is well known to induce both of these forms of cell death and the underlying mechanisms have been elaborately described. However, cells can also engage apoptosis and necroptosis through TNF-independent mechanisms, involving, for example, activation of the pattern recognition receptors Toll-like receptor (TLR)-3 and -4, or zDNA-binding protein 1 (ZBP1). In this context, cell death signaling depends on the presence of receptor-interacting serine/threonine protein kinase 3 (RIPK3). Whereas RIPK3 is required for TNF-induced necroptosis, it mediates both apoptosis and necroptosis upon TLR3/4 and ZBP1 engagement. Here, we review the intricate mechanisms by which TNF-independent cell death is regulated by RIPK3.

## Introduction

Regulated cell death is a biological process with essential functions in organismal development, tissue homeostasis, and immune defense. Regulated cell death comes in many forms [1], the best-known being apoptosis, a non-lytic type of cell death. Lytic cell death, known as necrosis, was believed to be unregulated, often resulting from an insurmountable cellular insult. However, over the last two decades it has become clear that necrosis can also be regulated [2], and several forms of regulated necrosis have been described [1]. These include necroptosis [3], ferroptosis [4], NETosis [5], parthanatos [6], pyroptosis [7], and cuproptosis [8], which are all molecularly regulated by distinct intracellular mechanisms.

Necroptosis is a form of regulated necrosis that is mediated by the kinase Receptor Interacting serine/threonine-Protein Kinase-3 (RIPK3) which phosphorylates and thereby activates the pseudokinase Mixed Lineage Kinase-Like (MLKL) to execute cell death [9–16]. Originally, necroptosis was identified to be a consequence of tumor necrosis factor receptor (TNFR)-1 signaling [17,18]. Engagement of the TNFR1 by TNF leads to the formation of a large TNFR1-associated signaling complex (complex I) that functions to induce gene expression, mainly by activating nuclear factor kappa-light-chain-enhancer of activated B cells (NF-κB) signaling [19]. One component of this complex is RIPK1 [20], a protein that contains an N-terminal kinase domain (KD) required to mediate TNFR1-induced necroptosis, an intermediate domain that is involved in NF-κB signaling and harbors a RIP homotypic interaction motif (RHIM) that allows for the interaction with other RHIM-containing proteins [21], and a C-terminal death domain (DD) which facilitates its binding to TNFR1 [22]. RIPK1 is ubiquitylated by Cellular Inhibitors of Apoptosis Proteins (cIAPs) that are also present in the TNFR1-induced signaling complex [23], resulting in the formation of a complex that leads to NF-κB signaling [24–26]. However, when RIPK1 is deubiquitylated, i.e. as a result of dysfunctional cIAPs [24,27] it releases from the complex and can engage two forms of cell death, apoptosis [23,28] and necroptosis [17,18]. The DD of RIPK1 can associate with the DD of FAS Associated Death Domain (FADD) (complex IIa), which leads to the recruitment of the serine protease Caspase-8, the initiator caspase of extrinsic apoptosis [23,29]. Humans express the Caspase-8 paralogue Caspase-10, which can also interact with FADD upon activation of the death receptors Fas (CD95) or tumor-necrosis factor related apoptosis-inducing ligand (TRAIL) receptor and TNFR1 [30–32], although both enzymes may also have distinct functions [33]. However, since Caspase-10 is absent from rodents it has not been a major focus of investigation.

RIPK1 can also associate with the necroptosis-mediating protein RIPK3 [9,10,15]. Apart from the presence of a C-terminal DD in RIPK1, RIPK3 and RIPK1 are structurally similar, and both contain a kinase domain and RHIM. Homotypic RHIM:RHIM interactions between these proteins leads to auto-phosphorylation and activation of RIPK3 (complex IIb) [9,34]. RIPK3 subsequently oligomerizes into amyloid-like structures [34,35] and recruits and phosphorylates MLKL [13,14]. Activated MLKL then undergoes a conformational change, oligomerizes and localizes to membranes (including the plasma membrane), where it mediates membrane rupture and cell death known as necroptosis [11,12,14,36,37].

TNFR1-induced cell death is strictly regulated. TNFR1 ligation primarily induces NF-κB signaling, which leads to the expression of survival proteins including cellular FLICE (FADD-like IL-1β-converting enzyme)-Inhibitory Protein (cFLIP), an enzymatically inactive Caspase-8-like protein that regulates cell death [38]. Humans express three cFLIP isoforms, long (cFLIP_L_) and two short isoforms named cFLIP short (cFLIP_S_) and cFLIP raji (cFLIP_R_), whereas rodents only express cFLIP_L_ and cFLIP_R_ [39]. All isoforms contain two death effector domains (DED), which mediate binding to Caspase-8 via DED interactions. Heterodimer formation of Caspase-8 and either cFLIP isoform results in the inhibition of apoptosis since Caspase-8 is unable to form fully matured dimers that are able to cleave (and thereby activate) apoptosis effector proteins [40,41]. However, cFLIP_L_-Caspase-8 heterodimers retain proteolytic activity, although substrate specificity between Caspase-8 homodimers and cFLIP_L_-Caspase-8 heterodimers appear to slightly differ [42]. Upon formation of complex IIa, Caspase-8 can cleave RIPK1 [43–47] and RIPK3 [48] to prevent necroptosis [49]. Caspase-8 can also cleave CYLD to prevent RIPK1 de-ubiquitylation and cell death [50]. Inhibition of Caspase-8 activity, pharmacologically [51] or upon infection by certain pathogens [52], therefore promotes TNFR1-induced necroptosis. Mice and humans with mutations in the Caspase-8 cleavage site in RIPK1 display embryonic lethality (mice) [44,46] or severe neonatal inflammation (humans) [53]. Engagement of TNFR1 thus primarily results in the activation of (pro-survival) gene expression pathways, but under certain circumstances can result in apoptotic or necroptotic cell death.

**Figure 1.**
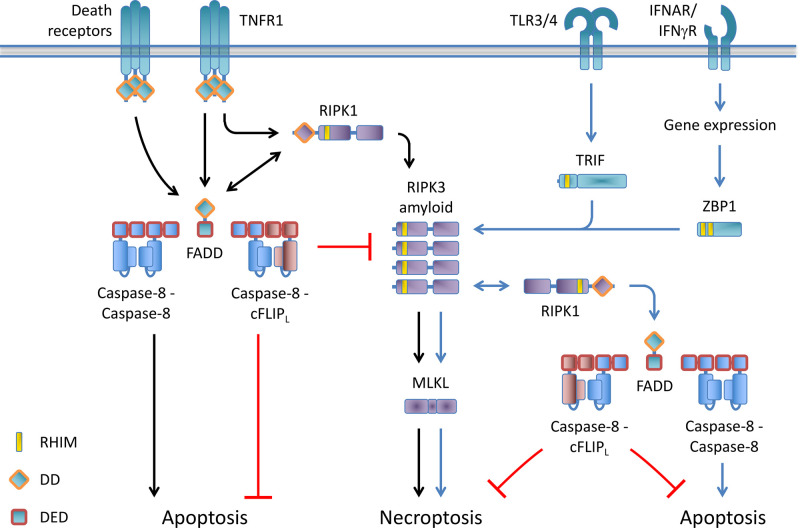
Pathways leading to extrinsic apoptosis and necroptosis. Upon engagement of death receptors such as TRAIL-R, CD95, and TNFR1, FADD recruits caspase-8 and cFLIP to regulate apoptosis. Caspase-8 homodimerization mediates apoptosis, whereas caspase-8 — cFLIP heterodimerization impairs apoptosis. TNFR1 engagement also results in RIPK1-mediated cell death. RIPK1 can engage with FADD to regulate apoptosis and RIPK3 to mediate necroptosis. RIPK1 — RIPK3 activation is controlled by the FADD — caspase-8 — cFLIP complex. Activation of TRIF-dependent TLRs or ZBP1 mediates cell death through association with RIPK3 and RIPK1. RIPK3 recruits MLKL to mediate necroptosis, which is controlled by the RIPK1 — FADD — caspase-8 — cFLIP complex. RIPK3 can mediate apoptotic signals to FADD — caspase-8. Type I and type II interferons induce the gene expression of ZBP1. Black arrows indicate death receptor-mediated pathways. Blue arrows indicate non-death receptor-mediated pathways.

The mechanisms of TNFR1-induced apoptosis and necroptosis have been well described (Figure 1). However, these cell death pathways can also be engaged by other, TNF-independent signaling cascades (Figure 1). Engagement of the adapter TIR-domain-containing adapter-inducing interferon-β (TRIF) by Toll-like receptor (TLR) 3 and 4, or the induction of the intracellular receptor zDNA-binding protein-1 (ZBP1, also known as DAI and DLM-1) in response to interferons can result in apoptosis and necroptosis, both of which depend on the presence of RIPK3. In this review, we focus on TNF-independent apoptotic and necroptotic cell death mechanisms in which RIPK3 plays a central role.

## RIPK3 can mediate death independently of RIPK1

TNFR1-induced necroptosis requires RIPK1 to engage RIPK3 (see above). Although resistant to TNF-induced necroptosis, *ripk1*-deficient cells show hyper-susceptibility to cell death induced by Poly(IC) (TLR3 ligand) or type I and type II interferons [54]. Moreover, these cells can undergo RIPK3-dependent cell death upon treatment with lipopolysaccharide (LPS, TLR4 agonist) [55], oxidized LDL (which is recognized by TLR4) [56], or upon certain viral infections [57,58]. In this setting, cell death is dependent on the presence of RIPK3 and mainly activates necroptosis. Cell death thus can ensue upon non-TNF-mediated activation of RIPK3. RIPK1, enigmatically, promotes TNF-induced necroptosis but prevents death when necroptosis is induced via alternate mechanisms [22] (Figure 1).

*Ripk1*-deficient mice die within a few days after birth [59]. Co-ablation of *ripk3* partially extends the life of *ripk1^−/−^* mice to ∼1 week, and these animals show apoptotic lesions in their intestinal tissue [54]. Co-ablation of *fadd* or *casp8* does not prevent the perinatal lethality of *ripk1^−/−^* mice. However, the combinatory ablation of FADD-Caspase-8-mediated apoptosis and RIPK3-MLKL-mediated necroptosis prevents organismal death, and the resulting animals reach adulthood [54,60]. Although RIPK1 kinase-deficient (*ripk1^K45A^*) mice are viable, mice in which the RHIM of RIPK1 is rendered dysfunctional (*ripk1^mRHIM^*) die in a similar fashion to *ripk1^−/−^* mice, and display signs of necroptosis in various tissues [61,62]. Unlike *ripk1^−/−^* mice, however, early lethality of *ripk1^mRHIM^* mice does not depend on Caspase-8-mediated apoptosis and can be prevented by ablation of *ripk3*, *mlkl*, or by introducing kinase inactivating or RHIM mutations in *ripk3* (*ripk3^D161N^* or *ripk3^mRHIM^*, respectively) [61,62]. These genetic data indicate that newborn mice encounter signals that activate receptors which are able to induce apoptosis and necroptosis mediated by FADD-Caspase-8 and RIPK3-MLKL, respectively, and are inhibited by RIPK1 in a RHIM-dependent manner.

The presence of RIPK1 thus suffices to confer protection from Caspase-8-mediated apoptosis in early life. *Ripk1^−/−^* MEFs show a reduction in cellular levels of cFLIP upon TNF treatment, whereas *ripk1^mRHIM^* MEFs retain cFLIP levels [61]. Although not formally demonstrated, the suggested presence of cFLIP may explain why apoptosis is not observed in *ripk1^mRHIM^ripk3^−/−^* mice. These data indicate that TNF-induced Caspase-8-mediated apoptosis is partially responsible for the lethality of newborn *ripk1^−/−^* mice. However, ablation of *tnfr1* delays the lethality of *ripk1^−/−^* pups to ∼2 weeks, which indicates that mechanisms other than those induced by TNF activate RIPK3 *in vivo*. Co-ablation of *tnfr1* from *ripk1^−/−^ripk3^−/−^* mice significantly extends life with onset of death at several months [54]. These triple-deficient mice show signs of tissues undergoing apoptosis, which is in line with the observation that ablation of *casp8* or *fadd* prevents death of *ripk1, ripk3* double-deficient mice. It is plausible that in *ripk1, ripk3, tnfr1* triple-deficient mice, the FADD-Caspase-8 signaling cascade is activated by other death receptors such as Fas or TRAIL-R, although this has not been formally demonstrated.

## The RIPK3 necrosome

RIPK3 activation leads to the formation of a complex called the ‘necrosome’, a macromolecular amyloid-like structure that functions as a platform for MLKL activation. It forms upon the engagement of the RHIM of RIPK3 with other RHIM-containing proteins. RHIMs consist of a conserved tetrad sequence I(V) Q I(V) G that is flanked by hydrophobic sequences [63]. TNFR1-activated RIPK1 induces the formation of a RHIM-mediated RIPK1–RIPK3 hetero-amyloid [21], which forms the basis for the necrosome. The structure of the RIPK1–RIPK3 hetero-amyloid has been elucidated by several groups [63–67] and consists of a pair of parallel β sheets that come together in an antiparallel fashion [64], where the RHIMs of human RIPK1 (IQIG) and RIPK3 (VQVG) alternately stack to create the hydrophobic core of the hetero-amyloid [65]. This leads to the recruitment of additional RIPK3 molecules and growth of the amyloid structure. Whether hetero-amyloids of RIPK3 and non-RIPK1 RHIM-containing proteins form a similar structure remains to be determined.

Mutating residues at the C-terminal flank of the RHIM domain in human RIPK3 (N464 and M468, but not N464 alone) to aspartates does not affect RIPK3 amyloid formation but prevents RIPK3 auto-phosphorylation, MLKL phosphorylation and necroptosis [64,67]. However, RHIM-mutant RIPK3 (VQVG → AAA), which is unable to form amyloids, retains the ability to phosphorylate MLKL but cannot induce MLKL auto-oligomerization, suggesting that the RIPK3 amyloid is essential for MLKL oligomerization but not for MLKL phosphorylation [66]. Thus, RHIM engagement induces RIPK3 to assemble into amyloid-like structures, providing a platform for the recruitment, phosphorylation and oligomerization of MLKL.

Only four human proteins, and several viral proteins [52], are known to contain functional RHIMs: RIPK1 [21], RIPK3 [21], TRIF [68,69], and ZBP1 [70]. TRIF is an adaptor protein that mediates immunological responses to invading pathogens upon ligation of TLR3 and -4, whereas ZBP1 is an intracellular receptor that recognizes Z-form nucleic acids of endogenous and pathogenic origin and is induced by interferon signaling.

## TLR3/4-induced TRIF-mediated cell death

TLR signaling is dependent on the recruitment of the adapter proteins Myeloid differentiation primary response 88 (MyD88) and/or TRIF to the activated TLR. Except for TLR3, all TLRs signal via MyD88. TLR3 signaling solely relies on TRIF, whereas TLR4 signaling can be mediated by both MyD88 and TRIF [71], with the latter additionally requiring the adaptor protein TRAM [72]. MyD88 does not contain a RHIM and cannot engage cell death directly. However, ligation of TLR2, TLR5 or TLR9 can lead to necroptosis via an indirect mechanism where MyD88-mediated NF-κB and MAPK pathway activation results in the expression of TNF which, in turn, can induce TNFR1-mediated cell death [73].

TRIF can mediate cell death upon TLR3 or -4 activation (Figure 1). It contains a RHIM through which it can interact with RIPK3 and RIPK1 [68,69]. Ligation of TLR3 or -4 can induce necroptosis in bone marrow-derived macrophages (BMDM) [55,73], fibroblasts [54,73], and microglia [74] when Caspase-8 is inhibited. RIPK1 and Caspase-8 inhibit necroptosis in this setting [54,73], indicating that the RIPK1-FADD-Caspase-8-cFLIP complex (see above) attenuates necroptosis engaged by the activation of TRIF-dependent TLRs. The resulting TRIF–RIPK3–RIPK1–FADD-Caspase-8 complex can mediate apoptosis when the kinase function of RIPK3, and thereby necroptosis, is inhibited (see below) [75]. Thus, cell can undergo TRIF–RIPK3-mediated cell death upon sensing of pathogenic ligands by TLR3 and TLR4.

TRIF-mediated signaling leads to the activation of IRF3 to induce type I interferons [76], interferon-stimulated genes, and Erk1/2, cFos, and NF-κB signaling [69,77,78], which contribute to LPS-induced inflammatory responses *in vivo* [78]. Moreover, TRIF is required for the activation of type I interferon responses induced by the intracellular DDX1–DDX21–DHX36 RNA helicase dsRNA sensor complex in dendritic cells [79], and for signaling by STimulator of INterferon Genes (STING) [80]. As such, TRIF may play a role in the activation of cell death by interferons (discussed below).

## Interferons and cell death

Type I and type II interferons (IFNs) can induce cell death under conditions where caspases are inhibited or when *ripk1*, *fadd*, or *casp8* are deleted. This death is mediated by RIPK3 [54,81] and MLKL [60] and inhibited by the RIPK1-FADD-Caspase-8 complex [54]. These observations indicate that interferon-induced signaling can lead to RIPK3-mediated death.

However, the proteins that mediate the interferon signaling cascades do not contain RHIMs, and it was long unclear how interferons could induce necroptosis. It was therefore surmised that IFN signaling leads to the expression of genes involved in necroptosis. IFN induces expression of the RNA-responsive protein kinase R (PKR), which was suggested to engage with RIPK1 to induce the necrosome and execute necroptosis [82]. However, this finding is contradicted by studies showing that PKR is not involved [81,83,84], leaving a role for PKR in interferon-induced necroptosis obscure.

Evidence that IFNs induce necroptosis through the expression of downstream genes comes from studies showing that IFN-β-induced necroptosis requires STAT1, STAT2, and IRF9 [81] and IFN-β- and IFN-γ-induced necroptosis requires JAK1/STAT1 signaling [83,84]. Type I IFN signaling induces the formation of the ST2/IRF9 complex (consisting of a STAT2 homodimer and IRF9) and the Interferon Stimulated Gene Factor 3 (ISGF3) complex (consisting of a STAT1–STAT2 heterodimer and IRF9), which binds to interferon-sensitive response elements (ISRE) and regulates expression of downstream genes [85]. Type II IFN signaling induces STAT1 homodimers that engage with Gamma interferon Activation Site (GAS) elements to regulate the expression of downstream Interferon Stimulated Genes (ISGs) [85]. Interferons therefore were deemed likely to induce the expression of an ISG containing a RHIM. Indeed, both type I and type II interferons induce the expression of the interferon responsive gene ZBP1 [83,86,87], a cytosolic receptor that contains two RHIMs. ZBP1-deficient cells are completely protected from IFN-induced necroptosis [83,84], indicating that IFNs up-regulate the ISG ZBP1 to induce RIPK3-mediated cell death.

## ZBP1-induced cell death

ZBP1 is a pattern recognition receptor that detects Z-form nucleic acids of endogenous or viral origin [88–94]. It contains two N-terminal tandem Zα domains to recognize nucleic acids [90], followed by two putative RHIM through which it can interact with RIPK3 and RIPK1 [70,87,95], and a C-terminal domain that can recruit TBK1 and IRF3 to induce IFN-β expression and activation of the type I interferon pathway [96].

Although the exact origins of the endogenous ligands that activate ZBP1 remain obscure, deletion or inhibition of the only other Zα domain-containing protein adenosine deaminase acting on RNA 1 (ADAR1) leads to the accumulation of Z-form RNA elements (Z-RNAs) and activation of ZBP1, implying that ZBP1 is activated by endogenous Z-RNAs that are otherwise repressed by ADAR1 [97,98].

ZBP1 can recognize cellular infection by several viral families, including orthomyxoviruses (influenza A virus (IAV) and influenza B virus) [93,99], herpesviruses (murine cytomegalovirus, herpes simplex virus 1 (HSV-1)) [100–102], poxviruses (vaccinia virus) [103], flaviviruses (Zika virus) [104], and β-coronaviruses such as SARS-CoV-2 [105]. Activation of ZBP1, i.e. upon infection with murine cytomegalovirus (MCMV) [102] or IAV [93,99], leads to the recruitment of RIPK3 via RHIM interactions which mediates cell death (Figure 1).

ZBP1 is essential for the control of IAV infections. When the ability of ZBP1 to induce cell death is impaired, viral titers may reach levels that are insurmountable for animals to survive. Indeed, both z*bp1*- and *ripk3*-deficient mice are hypersusceptible to lethal IAV infection [93]. Interestingly, however, mice in which necroptosis is ablated by deletion of *mlkl* can control IAV infection. Similarly, mice in which Caspase-8-mediated apoptosis is impaired (by a Caspase-8 mutation that renders Caspase-8 unable to auto-process and does not engage apoptosis but remains able to inhibit necroptosis) can also overcome infection with IAV. However, mice in which both apoptosis and necroptosis are ablated (i.e. *casp8* and *mlkl* double deficient mice are not able to control rising viral titers and succumb to infection [57,93,106]. This indicates that activated ZBP1 can induce apoptosis as well as necroptosis. Indeed, also in settings of HSV-1 infection ZBP1 can induce both modes of cell death [101].

RIPK1 was thought to bridge the signaling from RIPK3 to FADD-Caspase-8. However, in *ripk1*-deficient cells, induction of ZBP1 by Interferons leads to RIPK3-dependent MLKL-mediated necroptosis and can also result in RIPK3-dependent Caspase-8-mediated apoptosis [83], indicating that RIPK3 can signal to Caspase-8 in absence of the RIPK1-FADD interaction, although the underlying mechanisms remain to be elucidated.

Canonically, ZBP1 activation is mediated by its Zα domains. However, heat stress induces the heat shock transcription factor 1 (HSF1) to express ZBP1, which is then activated to induce RIPK3-dependent death in a manner that does not depend on its nucleic acid sensing ability [107]. Thus, ZBP1 can induce apoptosis and necroptosis in response to nucleic acid sensing as well as other, yet to be defined, mechanisms. Cell death depends on RHIM:RHIM domain interactions between ZBP1 and RIPK3, and do not require the presence of other (RHIM-containing) adapter protein such as TRIF [58], or RIPK1 [58,83].

ZBP1 was suggested to constitutively bind RIPK1 through RHIM interactions [87]. In settings where TLR4 is activated in combination with the inhibition of TGFβ-activated kinase 1 (TAK1), e.g. during infection with *Yersinia spp.* (see below), TRIF is activated and recruits ZBP1–RIPK1, which in turn leads to the recruitment of FADD-Caspase-8, and cells subsequently die by apoptosis and/or pyroptosis (see below) [87]. *Ripk3^−/−^* cells also die in this setting [87], indicating that RIPK3 may not be absolutely required for TRIF–ZBP1-induced death.

Thus, several signaling cascades have been described through which ZBP1 activation can lead to cell death. The precise mechanisms that determine the outcome of ZBP1-induced, RIPK3-mediated and RIPK3-independent death remain incompletely understood however and may depend on yet to be identified interactors or post-translational modifications (detailed below).

## TRIF and ZBP1 promote RIPK3 activation *in vivo*

Ablation of *ripk3* or *mlkl* prevents the early lethality of *ripk1^mRHIM^* mice (see above) [61,62]. But which receptors induce this RIPK3-mediated lethality? Ablation of *trif* does not prevent lethality of *ripk1^mRHIM^* pups, but ablating *zbp1* in *ripk1^mRHIM^* mice renders animals that are viable and live full lives [61], indicating that ZBP1 engages RIPK3 to trigger necroptosis in *ripk1^mRHIM^* mice.

The lethality of *ripk1^−/−^* pups is prevented by the combined deletion of *casp8* and *ripk3*, but not either one alone (see above). ZBP1 plays only a partial role in mediating pathology in *ripk1^−/−^casp8^−/−^* pups, since co-ablation of *zbp1* delays death to ∼3 weeks after birth [83]. Concomitantly, abrogation of the interferon pathways delays death of *ripk1^−/−^tnfr1^−/−^* pups, with animals now living up to one month [83]. TRIF also seems to mediate pathology, with *ripk1^−/−^tnfr1^−/−^* pups living significantly longer when *trif* is co-ablated [83]. These data indicate that both the TRIF- and IFN-ZBP1 pathways are activated *in vivo* to mediate RIPK3 activation. Indeed, co-ablation of *trif* and *zbp1* from *ripk1^−/−^casp8^−/−^* mice yield animals that are similar to *ripk1^−/−^casp8^−/−^ripk3^−/−^*mice [61]. Thus, TRIF and ZBP1 promote RIPK3 activation when RIPK1 is absent *in vivo*. These observations support the idea that RIPK1, TRIF, and ZBP1 may represent the only molecules that function to activate RIPK3, at least in the setting where Caspase-8 activity is compromised *in vivo*.

## The kinase function of RIPK3 regulates cell death

RIPK3 deficiency is fully compatible with life, and several vertebrate species thrive whilst having lost *ripk3* over the course of evolution [52]. Mice do express RIPK3 and experimental ablation of the gene does not affect development or spontaneous induction of abnormalities throughout life [108].

**Figure 2.**
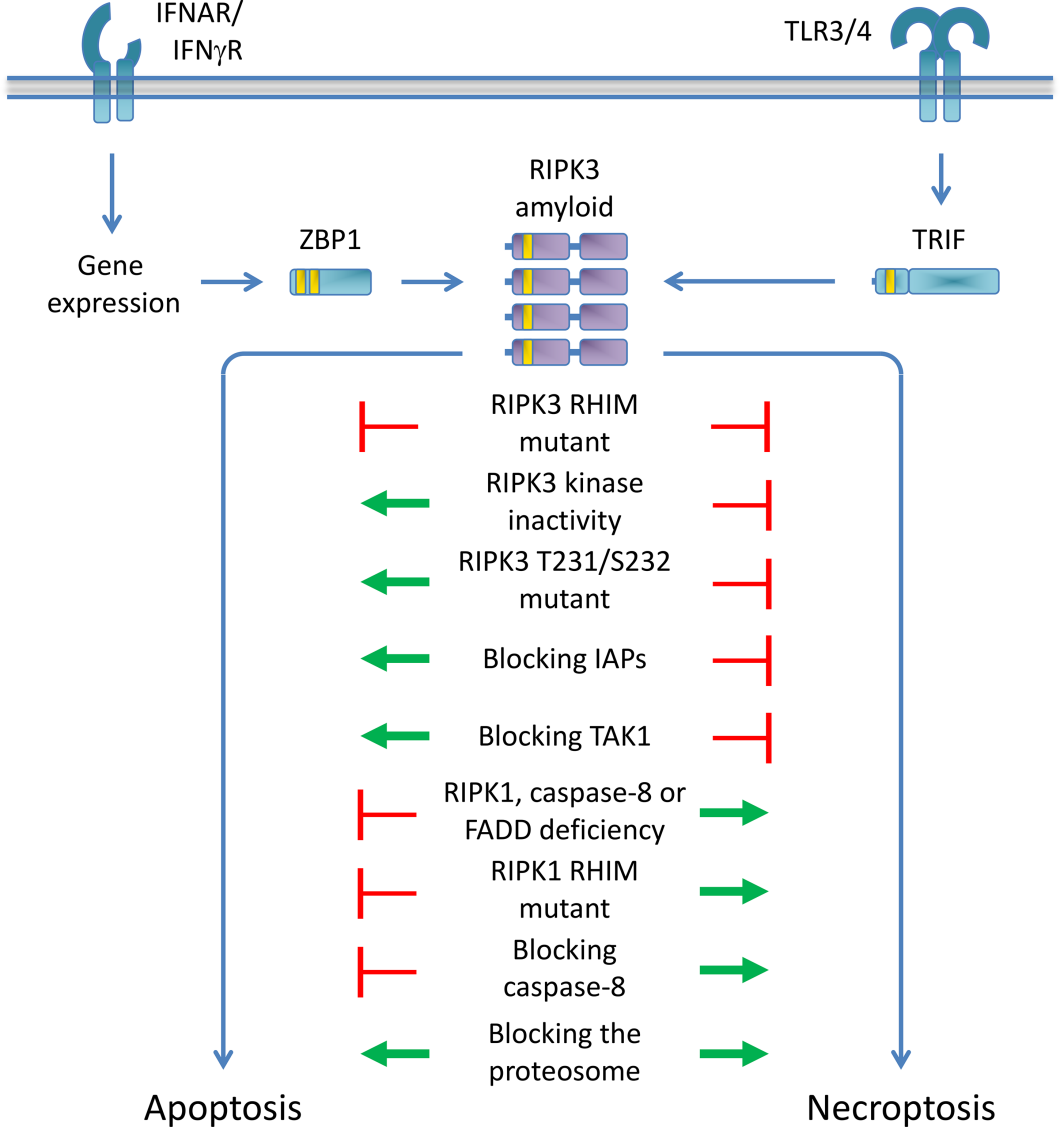
Regulation of non-TNF directed RIPK3-mediated death signals. Activated RIPK3 is able to induce both apoptosis and necroptosis. Disruption of the RIPK3 RHIM impairs both apoptosis and necroptosis. RIPK3 kinase inactivity or failure to phosphorylate RIPK3 T231/S232 impairs necroptosis but promotes apoptosis, a process that is furthermore regulated by cIAPs and TAK1. RIPK1, FADD, or caspase-8 deficiency, blocking the catalytic activity of caspase-8, or disruption of the RIPK1 RHIM promotes necroptosis and impairs apoptosis, although apoptosis can ensue under certain conditions in absence of RIPK1. Blocking the proteosome primarily promotes necroptosis, although apoptosis can also ensue.

The catalytic activity of RIPK3, however, can be vital to developing embryos and seems to influence death outcome (Figure 2). Catalytically inactive *ripk3^D161N^* mice die mid gestation due to mal-development of the yolk-sac [75,109]. Of note, the *ripk3^D161N^* mutation is unlike other mutations that render the kinase function of RIPK3 inactive and catalytically inactive *ripk3^K51A^* mice are viable [75]. Moreover, RIPK3 D161N does not act as a dominant negative mutant, since *ripk3^D161N/+^* mice are viable [109]. The mid gestational death of *ripk3^D161N^* mice is similar to the time of death of *casp8^−/−^* or *fadd*-deficient mice, which die by TNFR1-induced necroptosis of the yolk-sac vasculature [110–112]. The vascular endothelial cells of *ripk3^D161N^* yolk-sacs contain cleaved caspase-3, indicative of apoptosis [109]. This apoptosis is mediated by Caspase-8, and ablation of *casp8^−/−^* from *ripk3^D161N/D161N^* mice results in mice that reach adulthood and develop a phenotype similar to *casp8^−/−^ripk3^−/−^* mice [49,109]. However, how Caspase-8 is activated in *ripk3^D161N^* mice remains unclear and ablation of *tnfr1*, *mlkl*, *trif*, *zbp1*, *cyld*, *dr3*, or *cflar* does not prevent death of *ripk3^D161N^* mice [109]. Death does depend on RIPK1, which, when ablated, delays death from E11.5 to a few days after birth [109], similar to the lethality observed in *ripk1^−/−^* mice. Whether the combination of *trif* and *zbp1* or other, unidentified factors relay death signals through RIPK1 to Caspase-8 remains to be determined.

The precise mechanisms by which the kinase activity of RIPK3 functions in biology remains enigmatic. Blocking the RIPK3 kinase activity with the inhibitors GSK'843 or GSK'872 *in vitro* abrogates necroptosis and can induce the association of RIPK3 with RIPK1, FADD, Caspase-8, and cFLIP_L_ to mediate activation of caspase-3 and apoptosis, suggesting that the kinase activity of RIPK3, in part, determines cell death outcome [75,109]. But why would signals that induce apoptosis in catalytically inactive RIPK3 cells not cause necroptosis in wild-type RIPK3 cells? This may be due to the presence of a RIPK1-FADD-Caspase-8-cFLIP_L_ complex that blocks necroptosis. Why then does this complex not engage apoptosis in wild-type RIPK3 cells, or in cells where MLKL is absent? It has been suggested that the kinase and RHIM domains of RIPK3 collaborate to keep a conformation that controls association with apoptotic and necroptotic machineries [113], but unambiguous data is lacking.

One possible explanation may be that other proteins influence the ‘decisions’ of RIPK3. To be able to mediate necroptosis, RIPK3 must be correctly folded and is facilitated by the molecular co-chaperones heat shock protein 90 (HSP90) and Cell Division Cycle 37 (CDC37) [114]. Cells in which HSP90-CDC37 levels are high can undergo RIPK3-mediated necroptosis, whereas cells in which levels are low RIPK3 predominantly induces apoptosis [115]. In such HSP90-CDC37 low cells, two conserved serine/threonine residues (S164/T165 in human) in the kinase loop of RIPK3 are phosphorylated, possibly by RIPK3 itself, resulting in the abrogation of its kinase activity whilst potentiating the ability to recruit the RIPK1-FADD-Caspase-8 complex [115]. RIPK3 forms puncta in the cytosol upon necroptotic stimuli [10] but forms distinctive high-order structures in HSP90-CDC37 low cells [115]. The ‘decision’ of RIPK3 to relay a death signal to apoptosis or necroptosis therefore seems to depend on the structure of the signaling complex, which may be regulated by yet to be appreciated interactors.

Although the kinase activity of RIPK3 partly determines whether it induces apoptosis or necroptosis, the mechanisms by which RIPK3 controls cell death outcomes may be more subtle. RIPK3 engagement may also lead to either apoptosis or necroptosis in settings where the kinase activity is not impaired. Influenza A virus-derived Z-RNA activates ZBP1 (see above) in the nucleus of infected cells, which induces RIPK3-mediated death to control the infection [99]. IAV-infected individual cells die by either apoptosis or necroptosis [57,93,106], indicating that cells in which RIPK3 kinase activity is retained are able to undergo RIPK3-mediated apoptosis. It will be important to further elucidate how these intricate signaling cascades are regulated to fully understand the biology of RIPK3-mediated cell death.

## Post-translational modifications that influence apoptosis and necroptosis

RIPK-mediated cell death is strictly controlled by post-translational modifications, including ubiquitylation, phosphorylation, nitrosylation, PARylation, O-GlcNAcylation and acetylation.

RIPK3 can be S-nitrosylated at C119 by nitric oxide generated by neuronal nitric oxide synthase, mediated by N-Methyl-D-aspartic acid (NMDA) receptor, which affects neuronal damage during cerebral ischemia-reperfusion [116]. Upon TNF stimulation, poly-ADP-ribosylation (PARylation) of RIPK3 at C360 by tankyrase-1 leads to the recruitment of the E3 ligase RNF46 to complex II, resulting in the ubiquitylation and proteasomal degradation of complex II and inhibition of death [117]. O-linked β-N-acetylglucosamination (O-GlcNAcylation) of RIPK3 at T467 by O-GlcNAc transferase inhibits RIPK3s interaction with RIPK1 and other RIPK3 molecules, and necroptosis [118], whereas inhibition of sirtuins enhances RIPK1 acetylation at lysine residues K115, K625, K627, K642 and K648, and induces Caspase-8-mediated apoptosis engaged by the death receptor TRAIL-R [119].

Phosphorylation also plays a significant role in cell death or survival [34]. Auto-phosphorylation of RIPK1 and RIPK3 mediates necroptosis, but other kinases and phosphatases also affect RIPK-dependent cell death. For instance, the phosphatase Ppm1b dephosphorylates the T231/S232 residues of murine RIPK3 required for necroptosis [120] (Figure 2). TNF-mediated RIPK1-dependent cell death is inhibited by MK2, which phosphorylates RIPK1 at S321, thereby disrupting its ability to interact with FADD-Caspase-8 [121,122]. IKKα and IKKβ both directly phosphorylate RIPK1 at S25, inhibiting its kinase activity [123]. The kinase TAK1 functions upstream of the IKKs and help control whether TNFR1 ligation leads to gene expression or cell death. TAK1 kinase activity protects from TNF-induced RIPK1-dependent cell death [124–128]. Inhibition of TAK1, by the virulence factor *Yersinia* outer protein J (YopJ) expressed by *Yersinia* species bacteria or the pharmacological TAK1 inhibitor 5Z-7-Oxozeaenol (5z7), prevents the IKKα/β-mediated phosphorylation of RIPK1 at S25 and results in TNF-induced RIPK1-mediated cell death [123]. TAK1 activity is controlled by TAK1-binding protein 2 (TAB2) [129] and protein phosphatase 6 catalytic subunit (PPP6C) [130] and in their absence TAK1 is hyperactivated. However, where PPP6C deficiency prevents TNF-induced RIPK1-mediated necroptosis [130], abrogation of TAB2 leads to an exacerbation of TNF-induced necroptosis mediated by RIPK3 [127]. Moreover, deletion of TAB2 sensitizes PPP6C-deficient cells to necroptosis [130]. This indicates that TAB2 restricts necroptosis in a currently unappreciated manner.

TAK1 also regulates TLR4-mediated cell death (Figure 2). During *Yersinia* infection, YopJ inhibits TAK1 [131,132] whereas accompanying LPS triggers TLR4, resulting in a mix of apoptosis and pyroptosis in cell pools [133]. Treating cells with LPS and 5z7 mimics these effects [134]. When TAK1 is inhibited, LPS induces CD14-mediated TLR4 internalization, leading to the recruitment of TRAM-TRIF and a pre-formed ZBP1–RIPK1 complex. This induces RIPK1 phosphorylation and the recruitment of FADD-Caspase-8, resulting in Caspase-8 activation and cell death [87]. Death occurs in absence of RIPK3 or MLKL but is completely dependent on Caspase-8, which mediates cleavage (activation) of the apoptosis effector proteins caspase-3 and caspase-7, and the pyroptosis effector proteins gasdermin D (GSDMD) and gasdermin E (GSDME) [87,133,134]. The activity of TAK1 thus seems to control TLR4-induced signaling complex formation, the requirement for RIPK3 in the TLR4-induced death complex, and the mode of cell death this complex engages. The precise mechanisms remain unclear, but it will be interesting to elucidate which proteins are targeted by TAK1 to regulate RIPK3-dependent or -independent death. Moreover, it will be interesting to evaluate whether TAB2 also plays a role in restricting non-TNF-induced necroptosis.

Ubiquitylation also plays a significant role in cell death or survival. cIAP1/2-mediated ubiquitylation of RIPK1 keeps RIPK1 in the TNFR1 complex and impairs cell death [27,28]. The deubiquitylating enzymes Cylindromatosis (CYLD) also regulates RIPK1 ubiquitylation, but after RIPK1s release from the TNFR1 complex [135]. RIPK3 is also ubiquitylated upon TNFR engagement. K63-linked ubiquitylation of RIPK3 Lys-5, regulated by the deubiquitylase A20, is required for TNF-induced necroptosis [136]. cIAP1/2 and XIAP regulates the function of RIPK3 upon TLR4 engagement [137]. In the presence of these E3 ligases, LPS induces TRIF-cIAP-dependent ubiquitylation of RIPK3 and MLKL, allowing necroptosis to ensue (Figure 2). However, in the absence of cIAP1/2 and XIAP, LPS triggers RIPK3-mediated activation of Caspase-8, which results in apoptosis or activation of the NLRP3 inflammasome which does not depend on RIPK3s kinase activity or the presence of MLKL [137]. Thus, the ubiquitylation status of RIPK3 helps determine cell death outcome upon ligation of TLR4.

The ubiquitin-proteosome system regulates RIPK3-mediated necroptosis and can induce necroptosis without the activation of receptors. Blocking the proteosome leads to the accumulation of K48-linked ubiquitylated RIPK3 (at K264) and induces necroptosis (Figure 2). Death is dependent on an intact RHIM in RIPK3, suggesting that RIPK3 accumulation suffices to induce necroptosis. Death is not dependent on the presence of cIAPs and the E3 ligase responsible for the K48-linked ubiquitylation of RIPK3 K264 remains to be determined [138]. Another recently identified mechanism that can induce necroptosis without activating receptors is increase in intracellular pH [139], induced when the Na^+^/H^+^ exchanger SLC9A1 mediates a more basic intracellular pH in response to osmotic stress. This pH change activates RIPK3 and induces necroptosis, however without the requirement for its RHIM domain or RIPK1 [139]. This finding underscores that the exact mechanisms and settings by which RIPK3 mediates necroptosis remain incompletely understood.

## Concluding remarks

RIPK3-dependent cell death can be engaged by three currently known mechanisms, TNFR1, TRIF-dependent TLRs, and ZBP1, and can lead to apoptosis, necroptosis, and pyroptosis. Although the mechanisms by which RIPK3 is engaged and mediates cell death are becoming increasingly clear, recent insights indicate that many regulatory aspects of RIPK3-dependent cell death remain to be understood. Elucidating how these intricate signaling mechanisms work to control cellular demise will not only be important to fully understand the biology of RIPK3-mediated cell death but may also provide avenues for novel therapeutic strategies to treat the various human diseases that involve RIPK3.

## Abbreviations

ADAR1: adenosine deaminase acting on RNA 1
CDC37: Cell Division Cycle 37
CYLD: Cylindromatosis
DD: death domain
DED: death effector domains
FADD: FAS Associated Death Domain
HSP90: heat shock protein 90
HSV-1: herpes simplex virus 1
IAV: influenza A virus
IFNs: interferons
LPS: lipopolysaccharide
MLKL: Mixed Lineage Kinase-Like
PKR: protein kinase R
PPP6C: protein phosphatase 6 catalytic subunit
RHIM: RIP homotypic interaction motif
RIPK3: receptor-interacting serine/threonine protein kinase 3
TAB2: TAK1-binding protein 2
TLR: Toll-like receptor
TNFR: tumor necrosis factor receptor
TRAIL: tumor-necrosis factor related apoptosis-inducing ligand
ZBP1: zDNA-binding protein 1
